# INSoles To Ease Pressure (INSTEP) Study: a multicentre, randomised controlled feasibility study to compare the effectiveness of a novel instant optimised insole with a standard insole for people with diabetic neuropathy: a study protocol

**DOI:** 10.1136/bmjopen-2019-029185

**Published:** 2019-03-23

**Authors:** Richard Collings, Jennifer A Freeman, Jos Latour, Patricia Jane Vickery, Sam Glasser, Vasileios Lepesis, Doyo Enki, Joanne Paton

**Affiliations:** 1 School of Health Professions Faculty of Health and Human Sciences, University of Plymouth, Plymouth, UK; 2 Podiatry, Torbay and South Devon NHS Foundation Trust, Torquay, UK; 3 School of Nursing and Midwifery Faculty of Health and Human Sciences, University of Plymouth, Plymouth, UK; 4 Peninsula Clinical Trials Unit, Plymouth University, Plymouth, UK; 5 Medical Statistics, University of Plymouth, Plymouth, UK

**Keywords:** diabetic foot, primary care, qualitative research

## Abstract

**Introduction:**

Foot ulceration is a multifactorial complication of diabetes. Therapeutic insoles and footwear are frequently used to reduce elevated tissue pressures associated with risk of foot ulceration. A novel protocol using in-shoe pressure measurement technology to provide an instant optimised insole and house shoe solution has been developed, with the aim of reducing foot ulceration.

**Aim:**

This study aims to assess the feasibility of conducting a multicentre randomised controlled trial to compare the effectiveness of a novel instant optimised insole with a standard insole for people with diabetic neuropathy.

**Methods and analysis:**

This study is a participant and assessor blinded, randomised, multicentre parallel group feasibility trial with embedded qualitative study. Seventy-six participants will be recruited from three podiatry clinics and randomised to an optimised insole plus usual care (intervention group) or standard insole plus usual care (control group) using a minimisation by randomisation procedure by study centre and previous ulcer status. Assessment visits and data collection will be at baseline, 3 months, 6 months and 12 months. Feasibility and acceptability of the trial procedures will be determined in terms of recruitment and retention rates, data completion rates, intervention adherence and effectiveness of the blinding.

Assessment of the appropriateness and performance of outcome measures will inform selection of the primary and secondary outcomes and sample size estimate for the anticipated definitive randomised controlled trial. Clinical outcomes include incidence of plantar foot ulceration and change in peak plantar pressure. Twelve participants (four from each centre) and three treating podiatrists (one from each centre) will be interviewed to explore their experiences of receiving and delivering the intervention.

**Ethics and dissemination:**

The study was approved by the South-West Exeter Research Ethics Committee. Findings will be disseminated through conference presentations, public platforms and academic publications.

**Trials registration number:**

ISRCTN16011830; Pre-results.

**Strengths and limitations of this study**

- This study assesses the feasibility of undertaking a definitive robustly designed large-scale randomised controlled study.
- This study contributes to the limited literature on feasibility of reducing foot ulceration by insole and footwear provision for those at risk of diabetic foot ulceration.
- Qualitative aspects of this study will help inform future studies to optimise their acceptability to patients.
- The current study is not designed to find differences in outcomes.

## Introduction

Foot ulceration is a devastating multifactorial complication of diabetes. It is expected that 25% of people with diabetes will develop a foot ulcer at some point.^1^ Foot ulceration is a limb and life-threatening condition known to precede 80% of all diabetic lower limb amputations.^2^

It is estimated that 30% of people with diabetes have diabetic peripheral neuropathy, a primary risk factor for the development of foot ulceration.^3^ The forefoot region is most susceptible to foot ulceration, particularly in neuropathic feet absent of protective sensation, where plantar loads and tissue stress are increased.^4–6^ Therapeutic footwear and insoles are provided to offload and reduce harmful tissue stresses in people with diabetes.^7^ Guidelines for foot care for people with diabetes recommend the use of therapeutic footwear and insoles in the preventative management of those at risk of foot ulceration.^8^

The efficacy of offloading the diabetic, neuropathic foot through the use of therapeutic footwear and insoles varies considerably.^7 9–12^ This variability may be explained by different study designs, and a lack of consensus in prescriptions for therapeutic footwear and insoles between clinicians, clinics and services which are largely based on expert opinion and clinical experience.^13^ There are no studies or protocols to indicate the optimal features or efficiency of the different devices designed to improve outcomes.

The use of an objective approach to guide footwear and insole design to improve clinical outcomes has been suggested. The use of pedobarography to identify vulnerable areas and influence the position and type of footwear and insole modifications may offer a more optimised approach and improve offloading outcomes.^14^ Arts *et al* 15 and Waaijman *et al* 16 introduced modifications to therapeutic footwear and insoles guided by in-shoe pressure technology, both noting reductions in peak pressure of 6.7%–24% and 15%–23%, respectively, compared with premodification. Lin *et al* 17 used in-shoe technology to guide the defined removal of ‘plugs’ at sites of interest out of the insole and achieved 32% peak pressure reduction. However current protocols focus only on altering the distribution of pressure across the weight-bearing foot. As yet, no consideration has been given to the temporal aspect of gait. Specifically, that total contact area between foot to floor (and therefore insole function) is dependent on the phase of gait and gait style.

To our knowledge, this is the first protocol that uses pedobarography to categorise the temporal loading pattern of the foot according to gait style, combined with information from pressure patterns. This information informs the design of insoles to optimally reduce in-shoe pressure through the implementation of a simple standardised algorithm. The insole is manufactured and issued at the same appointment, avoiding detrimental delays. This protocol describes a feasibility study to assess the implementation of a novel insole design algorithm aimed at producing insoles which optimally reduce in-shoe peak pressure and subsequent ulceration risk in people with diabetes and neuropathy. Therefore, the purpose of this study is to assess if a definitive randomised controlled trial (RCT) using a novel protocol is feasible.

Following recommendations from the Consolidated Standards of Reporting Trials (CONSORT) collaboration^18 19^ this feasibility trial will allow operational experience to inform the conduct and final design of a definitive trial so that it can be successfully delivered with confidence.

## Objectives

The purpose of this feasibility study is to:

- Assess the feasibility and acceptability of the trial procedures comparing the delivery of a novel instant optimised insole with a standard insole for people with diabetic neuropathy.
- Select the most appropriate primary outcome measure and secondary outcome measures and inform the sample size calculation of the future RCT.
- Explore the experiences of participants receiving optimised instant insoles and Pulman house shoe, or flatbed cushioned inlay insole and Pulman house shoe, and podiatrists’ experiences of delivering the intervention. This information will fine-tune the smooth delivery of the intervention protocol to optimise participant engagement in terms of recruitment, insole and footwear adherence, and minimise loss to follow-up.

## Methods and analysis

### Trial design/setting

**Figure 1:**
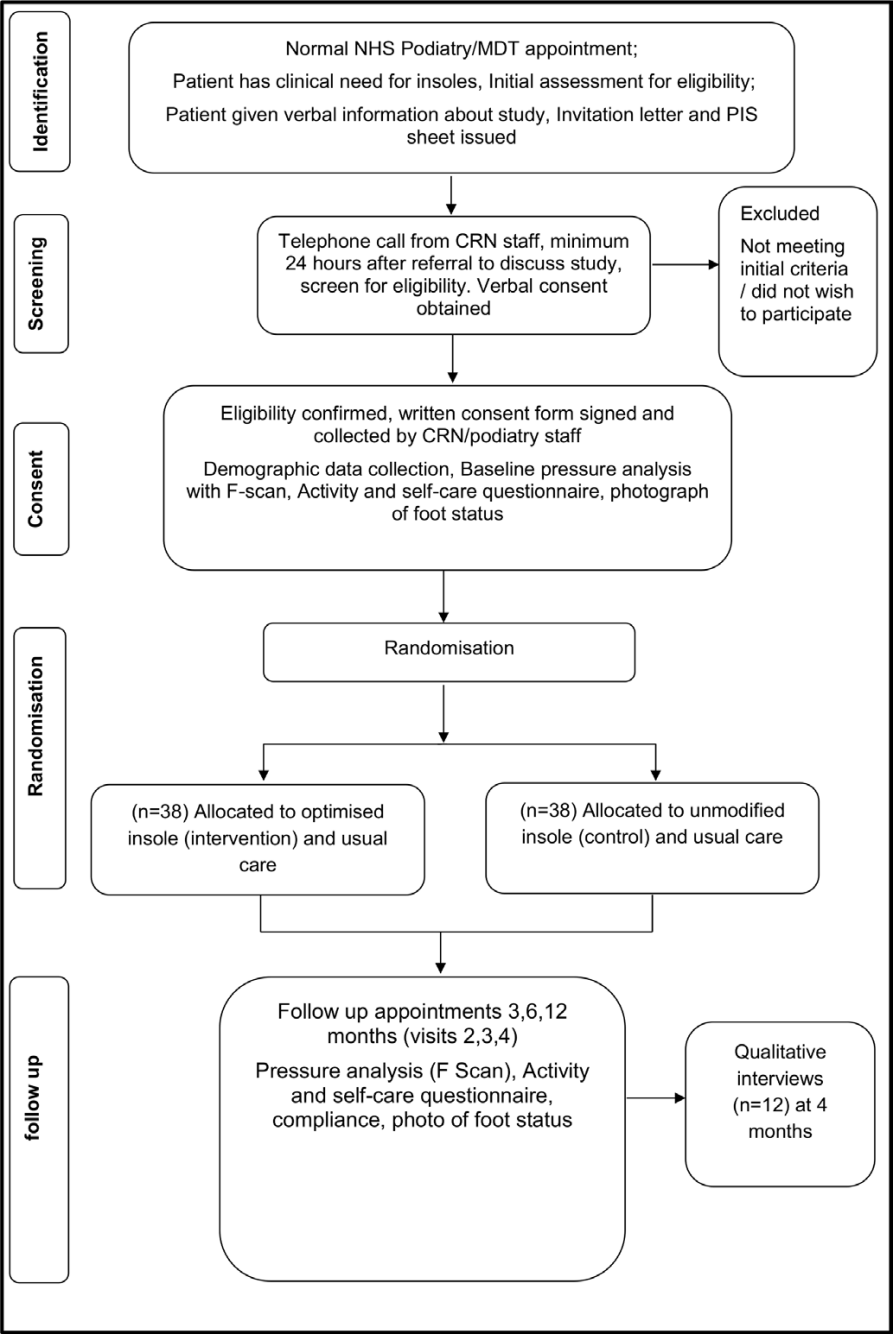
Trial flow chart. MDT, multidisciplinary diabetic team; NHS, National Health Service.

The Insoles to Ease Pressure (INSTEP) Study is a participant and assessor blinded, randomised, multicentre parallel group feasibility trial with an embedded qualitative study. A CONSORT Study flow chart is presented (figure 1) which outlines the flow of participants through the trial. Seventy-six participants will be randomised (allocation ratio 1:1) to receive an optimised insole plus usual care (intervention) or standard insole plus usual care (control). We will generate and implement the minimisation by randomisation procedure through a web-based system. This will ensure equal numbers of participants in the two groups by location, study centres and by previous ulceration status. Intervention group allocation (in a 1:1 ratio) will be revealed to the treatment podiatrist after the collection of baseline data. Insole and footwear will be issued by the same podiatrist immediately after randomisation. The allocated insole will be worn for 12 months. After initial baseline assessment, outcome measures will be attained at 3 months, 6 months and 12 months postrandomisation.

A subsample of 12 trial participants (4 from each centre) and three treating podiatrists (one from each centre) will be purposely selected. Semistructured interviews will be used to qualitatively explore their experiences of receiving/delivering the intervention. In addition, a further six participants will complete a daily journal with a 1-week account of their experiences at 3 months, 6 months and 12 months.

Trial centres are three community hospitals located in the south-west of England: Torbay (Torbay and South Devon NHS Foundation Trust), Exeter (Royal Devon and Exeter Foundation NHS Trust) and Solent (Solent NHS Trust).

### Participants and recruitment

People with diabetes and neuropathy will be identified and initially screened for eligibility by the usual podiatry clinical team, while attending for a routine foot care appointment located within the hospital-based multidisciplinary diabetic team foot clinic or podiatry community clinic. The inclusion and exclusion criteria are presented in table 1. Potential participants will be given a brief verbal explanation of the trial by their treating podiatrist and provided with written information. They will have at least 24 hours to review this information and ask any further questions before volunteering to participate. Potential participants will be telephoned to confirm their continued willingness to participate and offered an appointment for baseline measurement. Written informed consent will be obtained by a Good Clinical Practice trained nurse or podiatrist at the baseline visit.

**Table 1:** Insoles to Ease Pressure (INSTEP) Study inclusion and exclusion criteria

Inclusion criteria	Exclusion criteria
Confirmed diagnosis of type 1 or type 2 diabetes as confirmed by medical records	Any other significant disease or disorder*
Aged over 18 years	Non-healing foot ulcer at another site that requires targeted offloading
Identified clinical need for offloading insoles by podiatrist	Unable to walk 5 m with/without walking aid
Neuropathic (sensory peripheral diabetic neuropathy defined as insensitivity of a 5.07/10 g monofilament^16^)	Unable to stand on either leg independently for 10 s (±chair aid to assist in balance)
Palpable pedal foot pulses	Lacking capacity or unwilling to give consent
Able and willing to comply with all trial requirements	Already wearing existing insoles that are clinically appropriate
	Peripheral vascular disease (non-reconstructible vascular disease as determined by arterial duplex)
	Unwilling to wear therapeutic footwear
	Gross foot deformity, for example, Charcot foot or fixed rear foot deformity
	Unable to provide adequate consent to undertake the trial procedures
	Major amputation of part of the foot

*Which, in the opinion of the principal investigator (PI), may put the participant at risk of health deterioration, such as falls.

### Intervention

Two different insoles will be evaluated for feasibility and acceptability in this trial: instant optimised insole (intervention) and cushioned inlay insole (control). Both insoles will be custom-made to foot size and constructed using materials commonly used in the manufacture of insoles for people with diabetes. Each insole will be fitted into a Pulman house shoe, which will be measured to fit the participants’ foot. In addition, both insoles will have an activated data logger (Orthotimer, Algeos, Liverpool, UK) embedded into the insole to measure adherence.

#### Optimised insole

The instant optimised insole will be designed and optimised using the F-scan in-shoe pressure analysis system (Tekscan, Boston, Massachusetts, USA). A novel algorithm based on walking gait style (Mr Wobbly, Mr Stompee, Mr Propulsive) has been developed. The design and modification(s) will be informed by the treatment algorithm (online supplementary material 1). The optimised insole will consist of a preconstructed base (slim-flex, full-length, low-density, Shore A30, Algeos, UK). Regions of interest (ROIs) therein may be further formed to accommodate for prominent areas, with the addition of modifications that are designed to offload pressures from bony prominences in specific regions. These will be used to reduce peak plantar pressure values in conjunction with real-time pressure data from the F-scan system in the specific ROIs.

10.1136/bmjopen-2019-029185.supp1 Supplementary data

#### Control insole

The control group will receive a flatbed cushioned inlay insole (3 mm Poron 4000) with 5 mm medium density EVA heel lift cut to shoe size.

### Study procedures

An overview of the study procedures are outlined in table 2. All participants will be invited to attend the baseline visit and three further study appointments at 3 months, 6 months and 12 months postrandomisation. Data at all time points will be collected in case report forms (CRFs) by the trial team. All data will be entered into a secure database by the Peninsula Clinical Trials Unit.

**Table 2:** Study procedures for the Insoles to Ease Pressure (INSTEP) Study

Procedure	Baseline clinic (visit 1)	3 months* follow-up clinic (visit 2)	4 months qualitative follow-up*	6 months* follow-up clinic (visit 3)	12 months* follow-up clinic (visit 4)
Confirmation of eligibility	X				
Informed consent	X				
Demographics and history	X				
Plantar pressure in-shoe recording	X	X		X	X
Randomisation	X				
Intervention provision (including plantar pressure recording)	X				
Outcome measures (ulcer incidence; photographs, activity and self-care questionnaires)	X	X		X	X
Semistructured interviews (in participants’ homes)			X		
Journal entries		X		X	X
Serious adverse event recording		X	X	X	X

*Postrandomisation.

#### Baseline visit (visit 1)

At visit 1, written informed consent will be obtained on arrival. In addition, clinical information will be obtained from the patient and their medical notes including demographics (gender, age, height, weight, smoker/non-smoker, ethnicity), medical history (type 1 or type 2 diabetes, time since diabetes diagnosis, history of previous foot ulceration, current foot ulceration, other medical conditions), blood glycated haemoglobin levels and glomerular filtration rate. This information will be used to account for cofounding variables and analyses of differences and similarities between intervention arm groups.

### Data collection

Participants will perform in-shoe measurement tests with the treating podiatrist. The F-Scan in-shoe pressure measurement system (Tekscan, Boston, Massachusetts, USA) is capable of reliable and repeatable data collection.^20–22^ The F-scan system sensors are connected to a computer via a cuff unit and a 9.14 m long cable. Data are collected at a sampling rate of 50 Hz for 4 s. The TekScan software identifies pressures from 960 sensing locations on the plantar foot. Plantar pressures can be identified from individual samples or peak pressures can be identified over a total stance phase.

To optimise the accuracy and repeatability of the data collected within this study the following precautions will be incorporated within the data collection protocol. New sensors will be provided for each participant for each individual foot, labelled and used to collect data from that foot throughout the duration of the study. Participants will put on standard socks (20 denier stockings) and will be fitted with a standard house shoe (Markell Shoe Company, Yonkers, New York, USA). Any callus on the foot will be removed prior to fitting the socks and footwear, and prior to any recording.

Using a standardised protocol, participants will then be asked to undertake two test walks between chairs.^23 24^ This will allow the determination of usual gait velocity and for acclimatisation of the sensors. Between the chairs a premarked 5 m walkway with a little extra at each end to allow for deceleration and acceleration of gait will be used to determine gait velocity (m/s). This will be calculated by stopwatch recording of the time taken to pass between the marks. The walks will allow for the sensors to bed in and the temperature to reach equilibrium. Before each data collection session each patient will be weighed and each pair of insoles calibrated against body weight. Following calibration, if sensor saturation pressure exceeds 2000 kPa the sensor will be discarded. Calibration will be checked for within-foot and between-foot repeatability, and if excessive variation of ±10% is observed, the sensor will be recalibrated.

The test will consist of two runs initially. However, extra runs may be required if gait velocity is not consistent (a maximum of 5% deviation will be allowed). From each run, a minimum of three steps per foot is required to be analysed (excluding first and last steps of each run).

Using recorded F-Scan data, a maximum of three ‘Regions of Interest’ can be identified for each participant, where *ROI=mean peak pressure >350 kPa and/or is a recently healed ulcer site(s) or callus/corn formation.* In addition, identification of type of gait style (Mr Wobbly, Mr Stompee, Mr Propulsive) by analysis of the recorded F-scan pressure time curve and force time curve will occur.

#### Follow-up visits

Postrandomisation at 3 months (visit 2), 6 months (visit 3) and 12 months (visit 4) will occur. In-shoe pressure measurement testing, described in the baseline visit, will be repeated. Outcome measures will be collected at each visit. Participants who cease involvement in the study prior to the visits will be invited to report the reason. This will be optional.

### Outcome measures

#### Primary outcomes

The primary outcomes include feasibility and acceptability of the INSTEP Study. Quantitative and qualitative feedback will be obtained to identify the main determinants of experience and acceptance of the INSTEP trial, in particular the following measures and operational criteria:

- Assessing numbers of eligible participants from the target population.
- Assessing recruitment and retention rates of eligible participants through the trial.
- Assessing the willingness of participants to be randomised.
- Assessing the pragmatism of delivering the insole intervention in the proposed settings.
- Measuring variation and fidelity in the delivery of the intervention in each group. A fidelity checklist will evaluate the adherence by the treating podiatrists to the standardised protocol of intervention delivery.
- Assessing the completeness of data sets/outcome measures.
- Assessing the success of the blinding.

#### Secondary outcomes

Anthropometric measurements of height and weight will be attained at baseline. In addition, sensory neuropathy,^25^ visual acuity, clinician rated biomechanical foot and ankle status using validated clinical measures (foot posture index FP-6,^26^ ankle joint,^27^subtalar joint,^28^ first metatarsal phalangeal joint range of motion^29^) and clinician rated balance status (Romberg’s test)^30^ will be collected.

Clinical outcomes in the form of mean peak plantar pressure at ROI, in the standardised Pulman house shoe and measurements of plantar foot ulceration^31^ as measured by photograph following predefined assessment criteria^32^ will be assessed at baseline, 3 months, 6 months and 12 months. Adherence to wearing the insole and footwear (Pulman house shoe) as measured by a data logger (Orthotimer, Algeos, Liverpool, UK) will be recorded.

Patient self-reported outcomes will be assessed at baseline, 3 months, 6 months and 12 months. The Nottingham Assessment of Functional Footcare Questionnaire^33^ is an instrument that is used in routine care to identify those whose usual foot care might put their feet at risk of future ulceration. The International Physical Activity Questionnaire^34^ is an instrument for monitoring of physical activity and inactivity.

#### Blinding

Every effort will be made to ensure the participants and the assessor (chief investigator) remains blinded to treatment allocation until the end of the study. The chief investigator and the participants will complete a blinding assessment form after each measurement session to evaluate the effectiveness of the blinding. Successful blinding will be assessed using the Blinding Index.^35^

### Statistical analysis plan

A comprehensive statistical analysis plan (SAP) will be drafted prior to the final database lock; the SAP will be agreed with the trial steering committee (TSC) in the absence of a data monitoring committee. An extended CONSORT^18^ flow chart will be used to present descriptive data on screening, enrolment, intervention allocation, follow-up and assessment. It will also show any deviations from protocol, for example, participants receiving an ‘incorrect’ treatment. Descriptive data will be presented by the intervention group on baseline characteristics, for example, age, sex, type of diabetes.

#### Proposed primary outcome analysis

Analyses will summarise the feasibility outcomes: data from screening, recruitment and follow-up logs will be used to generate realistic estimates of eligibility, recruitment, consent and follow-up rates in the trial population. In addition, adherence data (eg, session attendance and insole/footwear adherence) will be used to contribute to the evaluation of the acceptability and concordance to the insole intervention. Completion rates will be estimated for each of the patient-reported and clinical outcome measures at each time point. All such estimates will be accompanied by appropriate CIs, to allow assumptions to be made in the planning of the definitive trial. The baseline characteristics of individuals lost to follow-up will be compared with those who complete the feasibility trial to identify any potential bias. Means and SDs arising from differences between the intervention and control arms to inform a power calculation for sample size estimate for the main RCT will be made.

Progression to a full trial will occur if the following minimum success criteria are achieved, or if there is reason to believe that suitable enhancements can be made to the full trial to ensure that any concerns are circumvented:

- 70% recruitment of the intended 76 participants within the 13-month recruitment window.
- 75% retention of participants within the 12-month trial period.
- 80% completion rate of primary and secondary outcome measures at baseline, 3 months, 6 months and 12 months.

#### Proposed secondary outcome analysis

Further analyses will summarise the proposed primary and secondary patient-reported and clinical outcomes at each time point. Descriptive statistics of the proposed primary and secondary outcomes will be produced, as appropriate for each measure for each trial arm. Interval estimates of the potential intervention effects, relative to control only, will be produced in the form of a 95% CIs, to ensure that the effect size subsequently chosen for powering the definitive trial is plausible, but no formal hypothesis testing will be undertaken of the feasibility data.

#### Qualitative analysis

Thematic analysis will be used for the analysis of the interviews and journals. This method includes a strategy for identifying themes and subthemes.^36^ The transcripts of the interviews and journal entries will be uploaded to the qualitative analysis program NVivo. To avoid individual bias, two researchers will independently read and code the transcripts. The codes will be formulated from the text fragments and will possibly be revised during the process of reading the transcripts. The two researchers will then discuss the results of the individual codes and try to reach consensus. After this, the codes will be reviewed and themes will be formulated.

Meaningful text fragments will be determined, as will codes (subthemes) and themes related to the trial objectives. Data extracts will be accompanied by narrative to elaborate why the extract is analytically interesting.

All participants will be anonymised and pseudonyms used to demonstrate different participants’ experiences. If any information is disclosed during the trial that could pose a risk of harm to the participant or others, the chief investigator, where appropriate, will report and act accordingly.

### Patient involvement

Patients were involved in the design and are currently involved in the conduct of this research. During the planning stage, priority of the research question, choice of outcome measures and methods of recruitment were informed by discussions with patients through a focus group session and two structured interviews. Patients form the membership of the independent TSC and of the trial management group. Once the trial has been published, participants will be informed of the results in a study newsletter suitable for a non-specialist audience.

### Data collection and management

Trial data collected at each centre by the research podiatrists and clinical research nurses will be recorded on trial-specific CRFs and will be considered the source data. The data manager will review the data being sent at regular intervals and report back to each centre if there is any discrepancy. The original completed CRF will be checked for completeness to ensure there are no missing items. Data will be entered into the database via a bespoke web-based data entry system encrypted using secure sockets layer (SSL).

### Sample size

As this is a feasibility trial the sample size is pragmatic and a power calculation is neither relevant nor possible. In this feasibility trial, while centres are likely to start recruitment in a staggered fashion, our overall target recruitment will be 2 per month per centre up to a total of 76 participants (38 per group). A CI approach has been used to establish feasible adherence rates. Based on an estimated completion rate of 75%, at least 75 patients are required. This is based on obtaining a 95% CI for a single proportion with a specified lower bound of the CI of 0.70 and a marginal error of 0.05. Data collected on proposed secondary outcomes will provide data on which sample size calculations can be performed in the future RCT.

### Adverse events

Adverse events (AEs) are, according to the definitions, any unfavourable or unintended event affecting patients in the study. In cases of prolongation of hospitalisation, death or significant clinical sequelae, these events are defined as serious AEs (SAEs), the occurrence of which will be informed to the study sponsor and the TSC at short notice. During protocol treatment, all deaths, all SAEs that are life-threatening and any unexpected SAE must be reported to the chief investigator using the SAE form within 48 hours of the initial observation of the event. In this trial, only those non-serious AEs associated with the lower limb, foot and mobility need to be reported. Safety aspects of the study will be monitored by the TSC, which will receive unblinded data for its judgement.

To standardise and optimise implementation of the intervention, and to further ensure the safety and well-being of research participants, all participants will be provided with standardised information on footwear and insole usage, how to increase wear time of the insoles, foot self-inspection and what to do in the event of a ‘foot attack’. This information will be reiterated at each appointment by the treating podiatrist. Participants will be advised to contact the treating podiatrist should any problems occur, in order that they can advise remanagement of these issues.

### Ethical issues

The protocol, V.1.0 (12/7/2017), was reviewed by the South-West Exeter Research Ethics Committee (REC) and was given a favourable opinion (REC ref 17/SW/0169) on 18 September 2017. Health Research Authority regulatory approval was given on 19 September 2017 and the study was adopted on the NIHR portfolio on 15 August 2017. Plymouth University is the sponsor of the study. The study will comply with the International Conference for Harmonisation of Good Clinical Practice guidelines and the UK Framework for Health and Social Care Research

Amendments to the protocol or study documents will be submitted to the REC and can only be implemented once approval has been obtained. Amendments will be tracked in the protocol and the version of the protocol will be updated.

### Dissemination plan and impact

It is the intention that the results of this study will be published in peer-reviewed journals and presented at national and international conferences. Authorship will be determined per internationally agreed criteria for authorship. Participant-level data will be available following publication of results on request. Results will be disseminated to the patient and public community via social media, newsletter articles and presentations at patient conferences and forums, led by the patient partners.

## Discussion

The proposed study will allow for all information collected providing important parameters to consider running a large-scale RCT and to identify potential constraints and possible solutions.

Current trends in the provision of insoles and therapeutic footwear are diverse for people with diabetes and neuropathy at risk of foot ulceration.^37^ A scarcity of evidence base for the appropriate design, modification and manufacture results in a lack of clear guidance for clinicians. As healthcare systems are also moving towards personalised medicine, the use of an in-shoe pressure measurement system and insole paradigm that will guide and personalise insoles and therapeutic footwear with no manufacturing delays has been developed.

The main limitations of the study are those characteristic of feasibility studies, the lack of power to present a statistically significant difference in outcomes. It may have a high dropout rate, so predictors of discontinuation should be assessed comparing characteristics of compliant patients with those who were lost to follow-up.

However, and despite the aforementioned limitations, the findings and outputs from the proposed feasibility study will take us closer to designing a future cost-effective trial in people with diabetes and neuropathy at risk of foot ulceration.

## Supplementary Material

Reviewer comments
