# Supplementary figures and images for: INSoles To Ease Pressure (INSTEP) Study: a multicentre, randomised controlled feasibility study to compare the effectiveness of a novel instant optimised insole with a standard insole for people with diabetic neuropathy: a study protocol
Source: BMJ Open. 2019 Mar 23;9(3):e029185. doi: 10.1136/bmjopen-2019-029185 (PMC6477388; doi:10.1136/bmjopen-2019-029185)

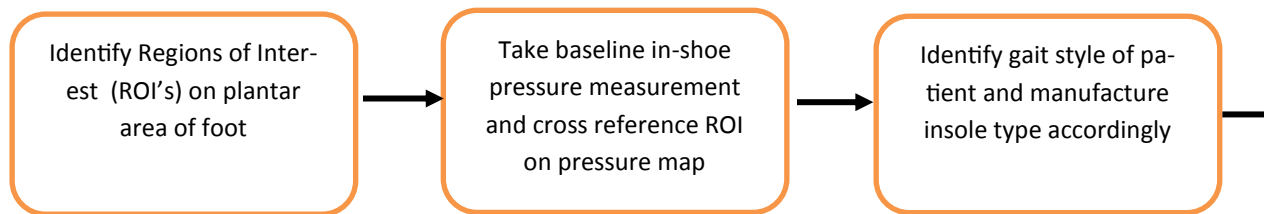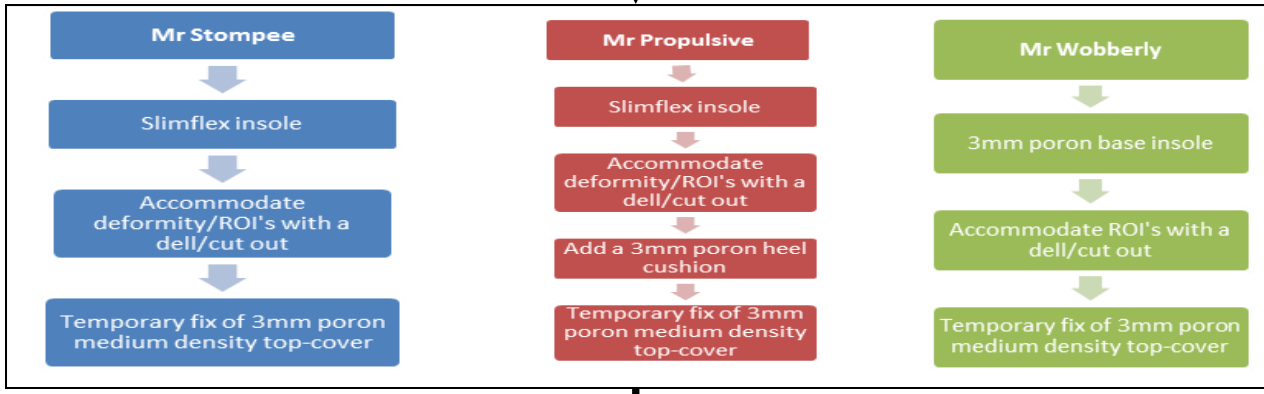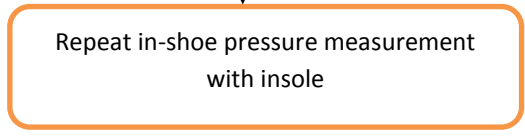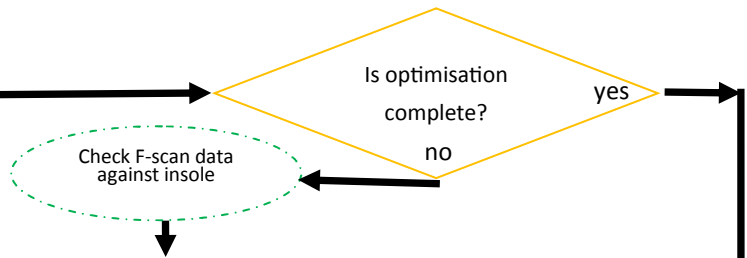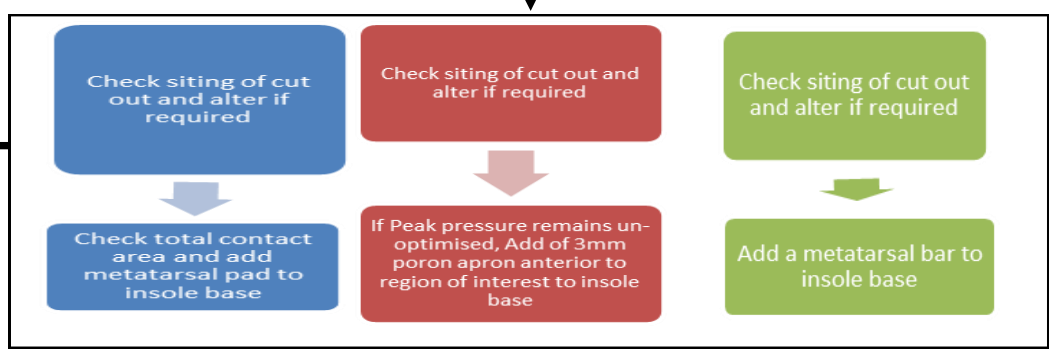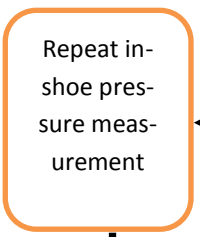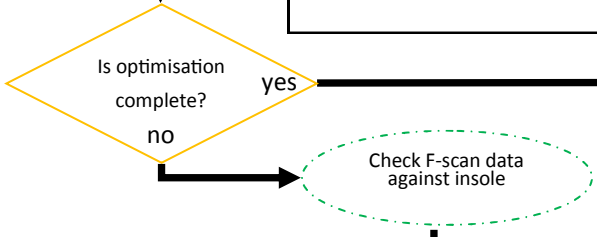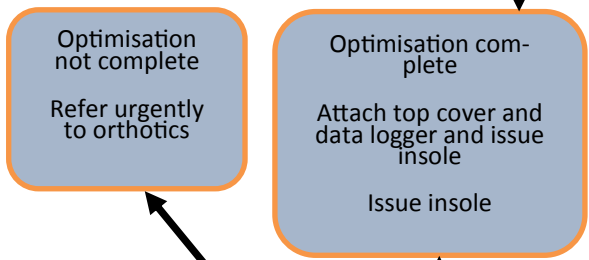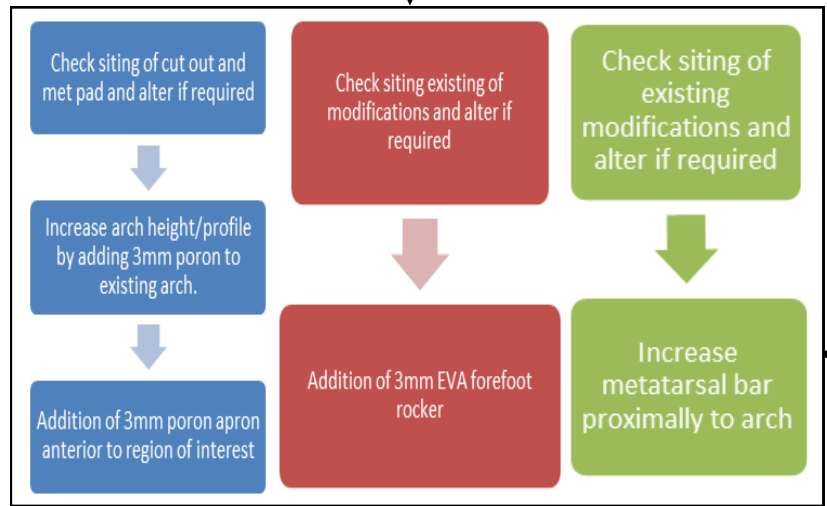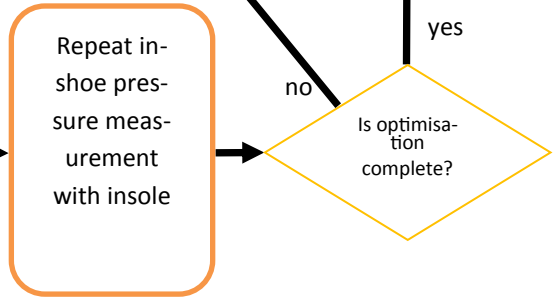

Supplement: Supplementary data [file bmjopen-2019-029185supp001.pdf]
