# Supplementary material for: INSoles To Ease Pressure (INSTEP) Study: a multicentre, randomised controlled feasibility study to compare the effectiveness of a novel instant optimised insole with a standard insole for people with diabetic neuropathy: a study protocol
Source: BMJ Open. 2019 Mar 23;9(3):e029185. doi: 10.1136/bmjopen-2019-029185 (PMC6477388; doi:10.1136/bmjopen-2019-029185)
Supplement: Reviewer comments [file bmjopen-2019-029185.reviewer_comments.pdf]

PEER REVIEW HISTORY

BMJ Open publishes all reviews undertaken for accepted manuscripts. Reviewers are asked to complete a checklist review form (**http://bmjopen.bmj.com/site/about/resources/checklist.pdf**) and are provided with free text boxes to elaborate on their assessment. These free text comments are reproduced below.

ARTICLE DETAILS

**TITLE (PROVISIONAL)**	INSoles To Ease Pressure (INSTEP): A multicentre, randomised controlled feasibility study to compare the effectiveness of a novel instant optimised insole with a standard insole for people with diabetic neuropathy: A study protocol
**AUTHORS**	Collings, Richard; Freeman, Jennifer; Latour, Jos; Vickery, Patricia; Glasser, Sam; Lepesis, Vasileios; Enki, Doyo; Paton, Joanne

This article was not externally reviewed at BMJ Open. Protocols that have been independently assessed prior to submission to BMJ Open are usually fast-tracked to publication on the grounds that further substantial changes will not be possible. This independent assessment will usually be external, independent review for both a substantial grant award from a non-commercial or government funder and ethics approval.
